# A Universal Digital Stress Management Intervention for Employees: Randomized Controlled Trial with Health-Economic Evaluation

**DOI:** 10.2196/48481

**Published:** 2024-10-22

**Authors:** Johanna Freund, Filip Smit, Dirk Lehr, Anna-Carlotta Zarski, Matthias Berking, Heleen Riper, Burkhardt Funk, David Daniel Ebert, Claudia Buntrock

**Affiliations:** 1 Department of Clinical Psychology and Psychotherapy Friedrich-Alexander-Universität Erlangen-Nürnberg Erlangen Germany; 2 School of Medicine and Health Technical University of Munich Munich Germany; 3 Department of Mental Health and Prevention Trimbos Institute Utrecht Netherlands; 4 Department of Epidemiology and Biostatistics Amsterdam University Medical Centers Amsterdam Netherlands; 5 Department of Clinical, Neuro and Developmental Psychology VU University Amsterdam Netherlands; 6 Department of Health Psychology and Applied Biological Psychology Leuphana University of Lueneburg Lueneburg Germany; 7 Division of eHealth in Clinical Psychology Department of Clinical Psychology Philipps University of Marburg Marburg Germany; 8 Department of Psychiatry VU University Amsterdam Netherlands; 9 Amsterdam Public Health Research Institute VU University Amsterdam Netherlands; 10 Institute of Information Systems Leuphana University of Lueneburg Lueneburg Germany; 11 Institute of Social Medicine and Health Systems Research Otto von Guericke University Magdeburg Magdeburg Germany

**Keywords:** economic evaluation, cost-effectiveness, cost-utility, cost-benefit, return-on-investment, employees, universal prevention, internet-based, stress management

## Abstract

**Background:**

Stress is highly prevalent and known to be a risk factor for a wide range of physical and mental disorders. The effectiveness of digital stress management interventions has been confirmed; however, research on its economic merits is still limited.

**Objective:**

This study aims to assess the cost-effectiveness, cost-utility, and cost-benefit of a universal digital stress management intervention for employees compared with a waitlist control condition within a time horizon of 6 months.

**Methods:**

Recruitment was directed at the German working population. A sample of 396 employees was randomly assigned to the intervention group (n=198) or the waitlist control condition (WLC) group (n=198). The digital stress management intervention included 7 sessions plus 1 booster session, which was offered without therapeutic guidance. Health service use, patient and family expenditures, and productivity losses were self-assessed and used for costing from a societal and an employer’s perspective. Costs were related to symptom-free status (PSS-10 [Perceived Stress Scale] score 2 SDs below the study population baseline mean) and quality-adjusted life years (QALYs) gained. The sampling error was handled using nonparametric bootstrapping.

**Results:**

From a societal perspective, the digital intervention was likely to be dominant compared with WLC, with a 56% probability of being cost-effective at a willingness-to-pay (WTP) of €0 per symptom-free person gained. At the same WTP threshold, the digital intervention had a probability of 55% being cost-effective per QALY gained relative to the WLC. This probability increased to 80% at a societal WTP of €20,000 per QALY gained. Taking the employer’s perspective, the digital intervention showed a probability of a positive return on investment of 78%.

**Conclusions:**

Digital preventive stress management for employees appears to be cost-effective societally and provides a favorable return on investment for employers.

**Trial Registration:**

German Clinical Trials Register DRKS00005699; https://drks.de/search/en/trial/DRKS00005699

## Introduction

In Europe, up to 27% of the working population suffers from stress [1]. Stress is often caused by work-related factors, including high perceived work demands, little work control, and little support from coworkers and supervisors [2]. Stress is linked to numerous diseases, including mental health problems and psychiatric diseases [3]. Besides the great burden of disease for the individual, stress is linked to formidable costs for employers as well as society as a whole. Due to stress and stress-related disorders, individuals suffer from impairment at work and lower productivity, are absent for more days from work, and use health services at a higher rate [4]. Lazarus’ transactional model of stress [5] delineates 2 distinct coping strategies. On the one hand, problem-focused coping involves actively influencing a stressful situation positively by using cognitive or behavioral efforts. On the other hand, emotion-focused coping primarily serves the purpose of managing challenging emotions, such as anger, disappointment, and sadness, in response to the specific situation.

The efficacy of stress management interventions has been confirmed in numerous meta-analyses of randomized trials in the general population [6] and in occupational settings [7]. However, high levels of psychological stress among employees are omnipresent and remain largely untreated [8]. Easily accessible and highly scalable digital interventions independent of time and place represent a promising approach to lowering the threshold for use compared with face-to-face interventions [9]. A recent meta-analysis provides evidence that digital or internet-based stress management interventions (iSMIs) are effective in terms of stress reduction in adults with small-to-medium effect sizes at posttreatment (Cohen *d*=0.43, 95% CI 0.31-0.51) [10]. In the workplace, in particular, a universal prevention approach is especially desirable because more employees can be reached without previous screening, which might be costly [11]. Compared with selective prevention with a focus on groups of people at increased risk or indicated prevention with a focus on individuals with elevated symptoms, universal prevention aims to reach the entire population regardless of any risk status [12]. A scalable digital intervention for the universal prevention of stress in the working population has the potential for substantial reach. Furthermore, with a focus on the entire working population, individuals who do not want to disclose symptoms due to fear of stigmatization can also be reached [13]. Concerning the efficacy of a universal iSMI for employees, the findings from a pragmatic randomized controlled trial indicate significantly reduced perceived stress with medium-to-large effect sizes both at posttreatment (Cohen *d*=0.71, 95% CI 0.51-0.91) and at 6-month follow-up (Cohen *d*=0.61, 95% CI 0.41-0.81) in the iSMI group when compared with a waitlist control condition (WLC) [14].

Yet, although the effectiveness of iSMI has been demonstrated, research on its economic merits is still limited. Evidence suggests that an indicated iSMI to proactively prevent the onset of stress in employees represents good value for money from both a societal and an employer’s perspective [15,16]. However, no study has yet evaluated the cost-effectiveness of a universal iSMI in the working population. This study, thus, aimed to evaluate the cost-effectiveness and cost-utility of a universal iSMI for stress in employees compared with a WLC from a societal perspective and the cost-benefit from the employer’s perspective over a time horizon of 6 months. The clinical effectiveness of this iSMI has already been established [14].

## Methods

### Study Design

We carried out the health-economic evaluation alongside a 2-arm randomized controlled trial (RCT), comparing the effects of a self-guided iSMI for stress prevention with a WLC. Detailed information about the study design can be found elsewhere [14]. We carried out and reported the health economic evaluation in accordance with the guidelines of the International Society for Pharmacoeconomics and Outcomes Research [17] and the Consolidated Health Economic Evaluation Reporting Standards statement [18] (more details in Multimedia Appendix 1).

### Recruitment

Recruitment was carried out as part of the occupational health program of a large German health insurance company (BARMER) in a way similar to the intended implementation of the intervention in routine practice in the future. The goal was to recruit individuals from the general working population, not just individuals insured by this health insurance company. This was done primarily through reports in the member magazine of the health insurance company and the insurance company’s occupational health and safety management staff, who informed the human resources departments of collaborating companies about their employees’ possibility to participate in the study. Interested individuals who completed a web-based screening questionnaire and met eligibility criteria were asked to fill out the informed consent form. To best reflect the routine conditions, the inclusion and exclusion criteria were reduced to a minimum. Individuals who were included (1) were 18 years and older, (2) were currently employed, and (3) had internet access and a valid email address. Exclusion criteria only included (1) a risk of suicide as indicated by a score of greater than 1 on the Beck Depression Inventory suicide item [14] or (2) any diagnosis with psychosis or dissociative symptoms (self-reported). If participants were excluded from the study, they were provided with information about alternative treatment options available in routine care.

Overall, 396 participants were included in the study, with 198 randomized to either the iSMI or WLC condition. All participants completed the assessment at baseline, while 313 participants (79%) provided data at 6-month follow-up. Study dropout was not statistically significantly associated with any sociodemographic characteristics or initial perceived stress level. The average participant was 41.76 (SD 10.09) years old, female (302/396, 76%), highly educated (285/396, 72%), and employed full-time (296/396, 75%) with a working experience of 17.58 (SD 10.36) years. Almost half of the participants worked in a management position (169/396, 43%).

### Randomization and Masking

#### Overview

Study participants were randomly assigned to the invention or control group in a 1:1 ratio by an independent researcher not otherwise involved in the study. Randomization took place using a computer-based random numbers table (Randlist) to ensure equal sample sizes for both conditions. Detailed information about the randomization procedure can be found elsewhere [14]. During the randomization process, the assignment was hidden from the participants as well as the researchers involved in recruitment and study administration. After randomization, participants were not blind to the study conditions due to the nature of the intervention.

#### Control Condition

In both study conditions, the participants had full access to treatment as usual (TAU). We did not interfere in TAU. Rather, we tried to maintain a naturalistic TAU state in order to represent routine care as much as possible.

#### Intervention

Participants in the intervention group (IG) received the iSMI GET.ON Stress. This iSMI entails 7 regular modules and 1 additional booster session for reviewing the most relevant content. Each module consists of psychoeducation, strategies for problem-solving, emotion regulation techniques, and plans for the future. It was recommended to complete 1-2 modules per week. The iSMI is based on the transactional model of stress [14]. The training includes interactive education, exercises, testimonials, and audio and video files. The content of the intervention is tailored to the individual needs and interests of the participants since several choices were made available through different answer options. In order to integrate the new knowledge sustainably into everyday life, homework, behavior planning, and an online diary are parts of the intervention. There was no therapeutic guidance provided. However, the participants had the opportunity to receive automatic text messages on their mobile phones. Participants could choose between light support with 1 text message every other day or intensive support with 2 or 3 text messages per day. The text messages contained very short exercises that should be carried out in everyday life to support the transfer from training to real life. More details about the intervention can be found elsewhere [14]. In this study, IG participants completed, on average, 5.23 (SD 2.74) sessions. Out of 198 participants, 94 (47%) participants finished all 7 modules, while 66 (33%) participants completed the additional booster session.

### Outcome Measurements

#### Health-Related Outcome

The health outcome in the cost-effectiveness analysis (CEA) was symptom-free status based on the Perceived Stress Scale (PSS) and defined as a score at a 6-month follow-up of 2 SDs below the baseline mean of the study population (22.65, SD 5.63) [19].

#### Quality-Adjusted Life Years

Quality-adjusted life years (QALYs) were used as a health outcome in the cost-utility analysis (CUA). QALYs were based on the 35-item version of the Assessment of Quality of Life (AQoL-8D), which was assessed at baseline and a 6-month follow-up and is a reliable and validated instrument [20]. In total, 8 dimensions of health-related quality of life were covered (ie, independent living, relationships, mental health, coping, pain, senses, self-worth, and happiness) and preference-based valuations of health states (utilities) on a scale of 0 (death) to 1 (perfect health) were generated, using the time trade-off method [21]. Cumulative QALYs gains over the study’s follow-up time of 6 months were estimated by calculating the area under the curve (AUC) of linearly interpolated AQoL-8D utilities between measurement points to cover the whole follow-up period.

#### Costs

##### Resource Use and Costing

We used the Trimbos and Institute for Medical Technology Assessment “Treatment Inventory of Costs in Patients with psychiatric disorders” (TiC-P) questionnaire to collect data on health care use, patient and family costs, and productivity losses [22]. The TiC-P is a retrospective questionnaire with a 3-month recall period and has been used in a similar study [15]. Costs were expressed in Euro and indexed from 2011 to 2013, the year the study was conducted, based on the German consumer price index (index factor 1.04) [23]. Costs were converted to pound sterling (£) using the purchasing power parities reported by the Organization for Economic Cooperation and Development [24]. For the reference year 2013, €1 was equated to £0.85 (A conversion rate of 1.33 in 2013 can be used for the conversion from € to US $).

##### Intervention Costs

At the time of conducting the study, the market price of the digital intervention provided by the GET.ON Institute, a commercial health care service provider was €99 (£84; ie, US $108.62) per participant, including costs for text messages, costs for website maintenance and hosting, technical support, and overheads.

##### Health Care Costs

We used 2 German guidelines for calculating health care costs [25,26]. Health care costs on a per-participant level were based on available lists of unit costs [26]. Unit costs were as follows: €20.92 (£17.78; ie, US $22.95) for a visit to the general practitioner, €46.55 (£39.57; ie, US $51.07) for a session with a psychiatrist, and €81.44 (£69.22; ie, US $89.36) with a psychotherapist, respectively. Costs per contact for allied health services (eg, physiotherapist) were valued at €17.08 (£14.52; ie, US $18.74). Hospital stays were computed at €335.52 (£285.19; ie, US $368.13) for an in-patient day in a psychiatric hospital and €306.41 (£260.45; ie, US $336.19) for an in-patient day in a hospital for psychosomatic medicine and psychotherapy. Costs were estimated by multiplying the units of resource use with corresponding unit costs. The costs of prescribed medication were based on Lauer-Taxe [27].

##### Patient and Family Costs

Out-of-pocket payments were directly obtained from participants. Costs for traveling were valued at €0.30 (£0.27; ie, US $0.33) per kilometer. Productivity losses from unpaid work (eg, household chores, shopping, and child care) and informal care were valued using the proxy good method (eg, price of a close market substitute: domestic help). The average gross wage of domestic help per hour was estimated at €18.33 (£15.58; ie, US $20.11) per hour.

##### Productivity Costs

Costs due to absenteeism (ie, days not worked) were valued according to the human capital method [28]. Lost working days due to absenteeism were valued at the gross average income of participants per day. Lost working days due to presenteeism (ie, reduced efficiency while at work) were computed by taking into account the number of working days for which the participant reported a reduced work performance weighted by an inefficiency score for those days (Osterhaus method) [29].

#### Statistical Analysis

##### Health-Related Outcome, QALYs, and Costs

Analyses were conducted according to the intention-to-treat principle using Stata (version 16; StataCorp) [30]. Missing data were imputed using multiple imputations by chained equations (MICE) using predictive mean matching to account for the skewed distribution of cost and utility data. The imputation model was stratified by the study arm and included demographic data (eg, age, gender, marital status, and education) alongside health-related outcome variables at baseline (eg, utility values and perceived stress). The number of imputed datasets was at least equal to the percentage of incomplete cases (ie, m=30). Analyses, as described below, were performed on each dataset separately, and results were pooled using Rubin’s rules.

##### Economic Evaluation: Societal Perspective

Disaggregated and total costs from the employer’s and societal perspectives, as well as QALYs per study group, were assessed with a set of ordinary least square regression equations, the latter adjusted for baseline utility values. Group differences in symptom-free status were tested using logistic regression.

From a societal perspective, the incremental cost-effectiveness ratio (ICER) was calculated as the extra costs per additional symptom-free participant or QALY gained, respectively. We bootstrapped seemingly unrelated regression equations (SURE) models to generate 2500 simulations of incremental cost and effect pairs while allowing for correlated residuals of the cost and effect equations and adjusting for potential confounders (eg, initial utility values in the effect equation). Based on the bootstrapped SURE models, bias-corrected and accelerated 95% CIs were obtained for incremental costs and effects. Bootstrapped cost and effect pairs were plotted on cost-effectiveness planes to graphically represent the uncertainty surrounding the ICERs. Cost-effectiveness acceptability curves (CEACs) were created to depict the probability of the intervention being cost-effective compared with the control condition for varying willingness-to-pay (WTP) thresholds.

To determine subgroups in which the intervention was particularly cost-effective, the net-benefit regression framework (NBRF) was used [31]. In the NBRF, the treatment dummy, prognostically relevant baseline characteristics, and their interactions are regressed on net benefit. Net benefit (NB) is defined as NB = (E*λ)–C, where E denotes the effects per participant (eg, QALYs), C denotes the costs per participant, and λ is the willingness-to-pay for a unit of effect (ie, €20,000 [ie, US $21,944] per QALY gained). Analyses were conducted using gender, age, education, marital status, work experience, previous health training or psychotherapy, level of perceived stress, resilience, agreeableness, psychological strain, and self-regulation competencies as independent variables.

##### Economic Evaluation: Employer’s Perspective

From the employer’s perspective, cost-benefit analyses were performed using 2 metrics: (1) net benefits (NB = benefits – costs; amount of money gained after costs are taken into account) and (2) return-on-investment (ROI = [(Benefits–Costs)/Costs] × 100%; percentage of profit per Euro invested), where costs are defined as intervention costs and benefits as the difference in productivity costs between the iSMI group and the control condition. The metrics were estimated by bootstrapping linear regression models (N=2500). The probability of a positive financial return was assessed by the proportion of positive estimates (eg, NB>0, ROI>0%).

##### Sensitivity Analyses

To test the robustness of the base case findings, we performed 3 sensitivity analyses. First, we applied Winsorizing, where extreme values of cost outliers (eg those above the 95th percentile) were replaced by the value at the 95th percentile. Second, we varied the costs of the intervention (ie, €299; ie, US $328.06) to reflect uncertainties about the actual market price. Third, we used another instrument, the EQ-5D-3L [32], to assess health-related quality of life instead of the AQoL-8D. While the first 2 sensitivity analyses were applied to both the societal and employer’s perspectives, the latter was only done from the societal perspective.

### Ethical Considerations

The study was approved by the ethics committee of the University of Marburg (Germany [Aktenzeichen 2014-30k (date: 08/14/2014]) and registered in the German clinical trials register (DRKS00005699) on December 12, 2014.

## Results

### Health-Related Outcomes and QALYs

Participants in the iSMI condition had a statistically significantly higher probability for symptom-free status at 6-month follow-up with an odds ratio of 5.14 (95% CI 3.23-8.18) compared with WLC based on bootstrapped data. The mean of the cumulative QALYs was higher in the iSMI condition (0.321 QALYs, 95% CI 0.32-0.33) compared with the WLC (0.306 QALYs, 95% CI 0.30-0.31). Adjusted incremental differences in QALYs between the iSMI condition and the WLC were statistically significant (Δ(e)=0.015 QALYs, 95% CI 0.01-0.02).

### Costs

Baseline costs were slightly higher in the iSMI condition (€2283, 95% CI 1916-2650 [US $2504.91, 95% CI 2102.24-2907.58]) but comparable to the WLC (€1936, 95% CI 1569-2303 [US $21.24.18, 95% CI 1721.51-2526.85]). The imputed mean 6-month cumulative per-participant costs (in €) separately for various cost categories by study condition are presented in Table 1. Direct medical costs, as well as patient and family costs, were similar in both groups. In the iSMI group, costs for absenteeism were higher than costs due to presenteeism. The opposite was seen in the WLC. Employer’s costs were slightly higher in the WLC compared with the iSMI (incremental difference of €76, 95% CI −667 to 515 [US $83.39, 95% CI –731.83 to 565.06]). Average total costs were comparable in both groups, with €3195 (US $3505.55; 95% CI 2661-3729 [95% CI 2919.65-4091.46]) in the iSMI condition and €3233 (US $3547.25; 95% CI 2722-3744 [95% CI 2986.58-4107.92]) in the WLC, resulting in an incremental difference of €38 (US $41.69; 95% CI −771 to 695 [95% CI –845.94 to 762.55]) in favor of the iSMI group.

**Table 1:** Imputed mean cumulative per-participant costs (in €) by condition over a 6-month follow-up period.

		Intervention group (n=198)			Control group (n=198)				Incremental difference			
		Mean, €^a^	95% CI	Mean, €		95% CI		Mean, €			95% CI	
**Direct medical costs**												
	Intervention costs	99	—^b^	—			—			99		—
	General practitioner	52	42 to 62	50			41 to 58			2		−11 to 16
	Mental health care	126	67 to 185	227			168 to 286			−101		−184 to −18
	Antidepressants	4	−5 to 14	15			5 to 25			−10		−24 to 4
	Allied health services^c^	41	23 to 60	30			13 to 46			11		−13 to 36
**Patient and family costs**												
	Over-the-counter drugs	20	13 to 28	26			19 to 34			−6		−16 to 5
	Out of pocket expenses^d^	106	56 to 156	104			61 to 148			2		−63 to 66
	Travel	11	6 to 16	15			10 to 20			−4		−11 to 4
	Unpaid work	159	90 to 229	187			122 to 252			−28		−125 to 70
	Informal care	330	195 to 466	194			92 to 297			136		−35 to 307
	Domestic help	168	89 to 248	133			44 to 223			35		−88 to 159
**Productivity costs**												
	Absenteeism	1217	897 to 1538	884			584 to 1184			334		−102 to 770
	Presenteeism	859	616 to 1103	1368			1128 to 1609			−509		−852 to −166
**Employer’s perspective**												
	Intervention costs + productivity costs	2176	1747 to 2605	2252			1841 to 2663			−76		−667 to 515
**Societal perspective**												
	Total societal costs^e^	3195	2661 to 3729	3233			2722 to 3744			−38		−771 to 695

^a^€1=US $1.10.

^b^Not available.

^c^For example, massage, physiotherapist, and occupational therapist.

^d^For example, allied health services without prescription.

^e^Includes all cost categories based on a bootstrapped (n=5000) linear regression model. Columns may not add up correctly due to rounding.

### Economic Evaluation

#### Societal Perspective

##### Cost-Effectiveness

**Figure 1:**
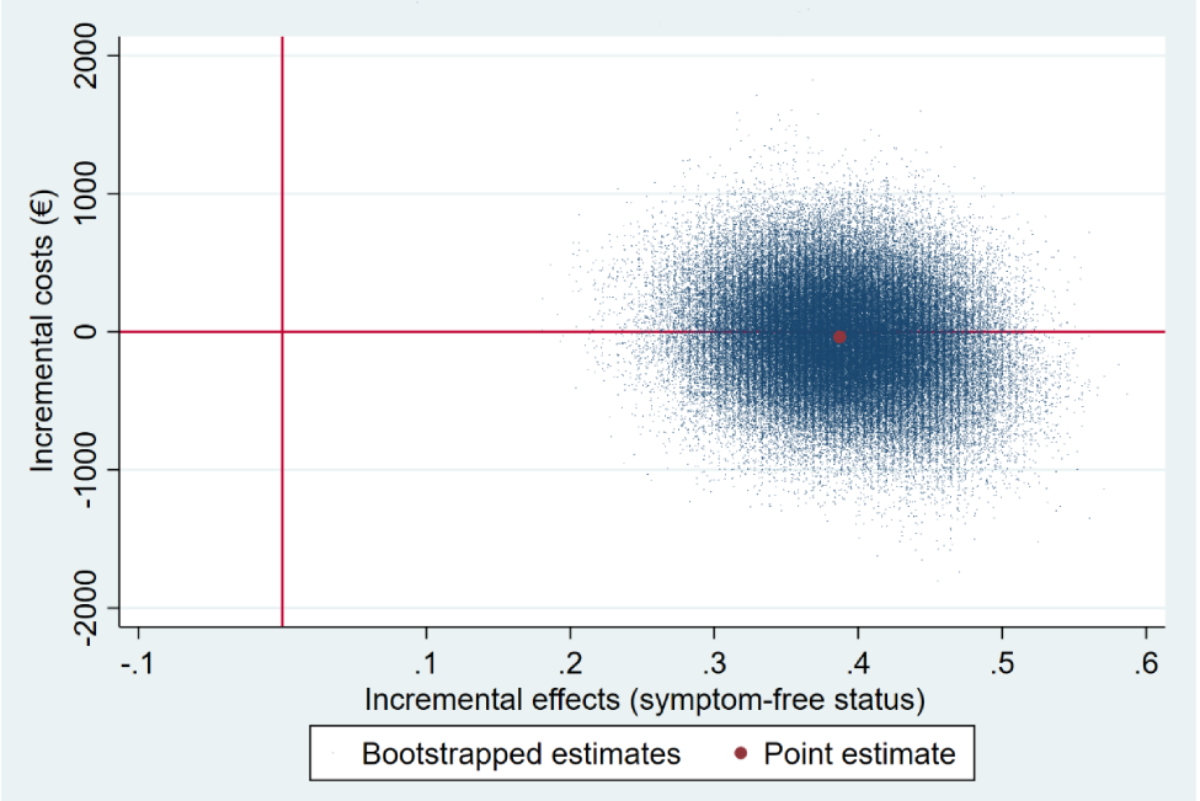
Scatterplot of 5000 replicates of the incremental cost-effectiveness ratio (mean differences in costs and symptom-free status) on the cost-effectiveness plane from the societal perspective: iSMI versus WLC.

**Figure 2:**
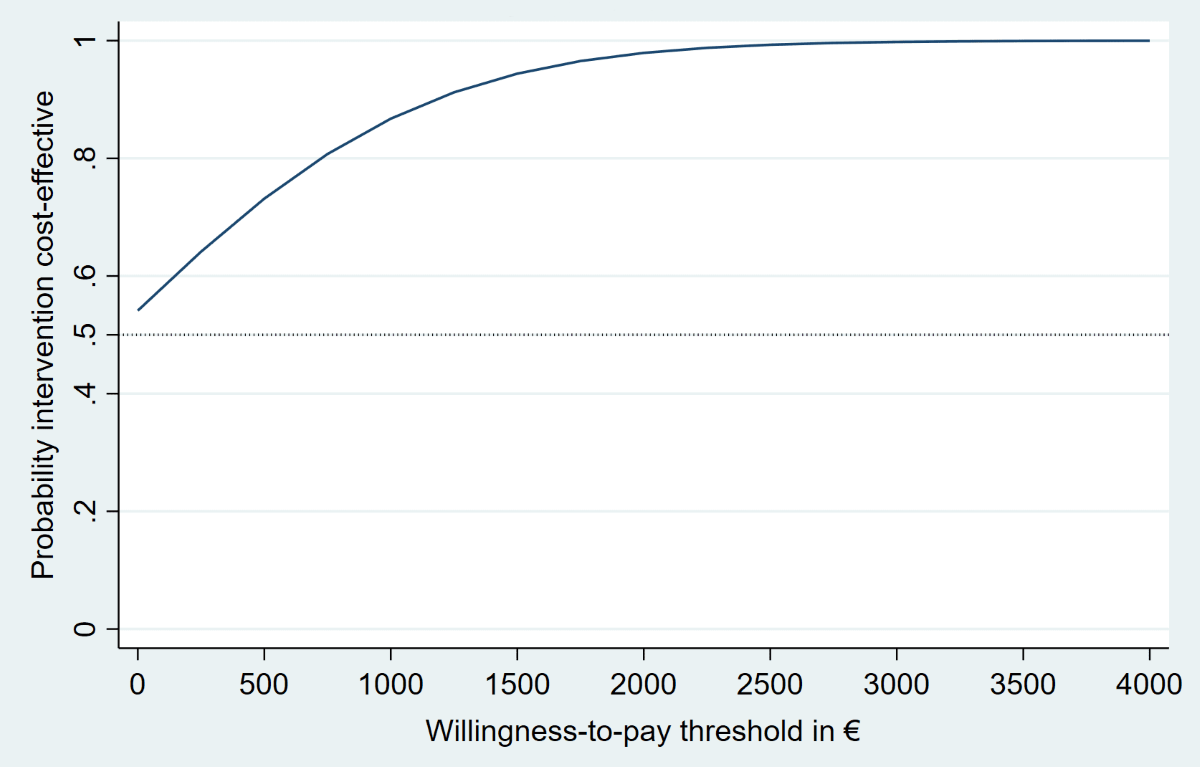
Cost-effectiveness acceptability curves (symptom-free status) from the societal perspective.

Table 2 shows the incremental costs, effects, mean ICERs, and the distribution of cost and effect pairs on the cost-effectiveness plane based on 2500 bootstrap simulations. Cost-effectiveness analysis revealed that the iSMI generated more symptom-free individuals (Δ[E]=0.39; 95% CI 0.29-0.48) at lower costs (Δ[C]=–€38 [£953; ie, US $1246.51]; 95% CI €–705 to €692 [ie, 95% CI US $–773.32 to $759.06]) relative to the WLC. With regard to the cost-effectiveness plane, 56% of the bootstrapped ICERs fell in the southeast quadrant, indicating a 56% probability that the intervention dominates WLC (Figure 1). The remaining 44% of ICERs fell in the northeast quadrant, demonstrating a 44% probability that the intervention leads to greater health gains but at higher costs than WLC. At a willingness-to-pay of €1500 (US $1645.37) per additional symptom-free person, the iSMI showed a probability of 94% being cost-effective compared with the WLC (Figure 2).

**Table 2:** Results from the societal perspective (main and sensitivity analysis) based on 2500 bootstrap simulations.

Outcome			Incremental costs, € (95% CI)		Incremental effects, points (95% CI)		Incremental cost-effectiveness ratio, € or points (95% CI)^a^		Distribution over the cost-effectiveness plane (%)						
									North-east quadrant^b^		South-east quadrant^c^		South-west quadrant^d^		North-west quadrant^e^
**Main analysis**															
	Symptom-free status (0/1)	−38 (−705 to 692)		0.39 (0.29 to 0.48)		Dominant		44		56		0		0	
	QALYs^f^ (range: 0-1)	−38 (−735 to 687)		0.015 (0.01 to 0.02)		Dominant		45		55		0		0	
**Sensitivity analysis 1^g^**															
	Symptom-free status (0/1)	−110 (−652 to 477)		0.39 (0.29 to 0.48)		Dominant		34		66		0		0	
	QALYs^f^ (range: 0-1)	−110 (−652 to 477)		0.025 (0.01 to 0.04)		Dominant		34		66		0		0	
**Sensitivity analysis 2^h^**															
	Symptom-free status (0/1)	162 (−505 to 892)		0.39 (0.29 to 0.48)		€418		89		11		0		0	
	QALYs^f^ (range: 0-1)	162 (−505 to 892)		0.025 (0.01 to 0.04)		€6,515		89		11		0		0	
**Sensitivity analysis 3^i^**															
	QALYs^f^ (range: 0-1)	−38 (−735 to 687)		0.011 (0.00 to 0.02)		Dominant		45		55		0		0	

^a^In accordance with ISPOR best practice guidelines on “Model Parameter Estimation and Uncertainty,” we do not report any negative incremental cost effectiveness. Ratios (ICERs) since they are meaningless. Instead, we use the term “dominant,” which means that the intervention has a higher impact and comparatively lower cost with the WLC.

^b^The northeast quadrant of the CE plane, indicating that intervention is more effective and more costly.

^c^The southeast quadrant of the CE plane, indicating that intervention is more effective and less costly.

^d^The southwest quadrant of the CE plane, indicating that intervention is less effective and less costly.

^e^The northwest quadrant of the CE plane, indicating that intervention is less effective and more costly.

^f^QALYs: quality-adjusted life years.

^g^Sensitivity analysis 1 analyses for winzorizing cost outlier to 95% percentiles.

^h^Sensitivity analysis 2 analyses adding intervention costs of €299 (US $327.98; instead of €99 [US $108.59]).

^i^Sensitivity analysis 3 analyses for EuroQol for quality-adjusted life years.

##### Cost-Utility

**Figure 3:**
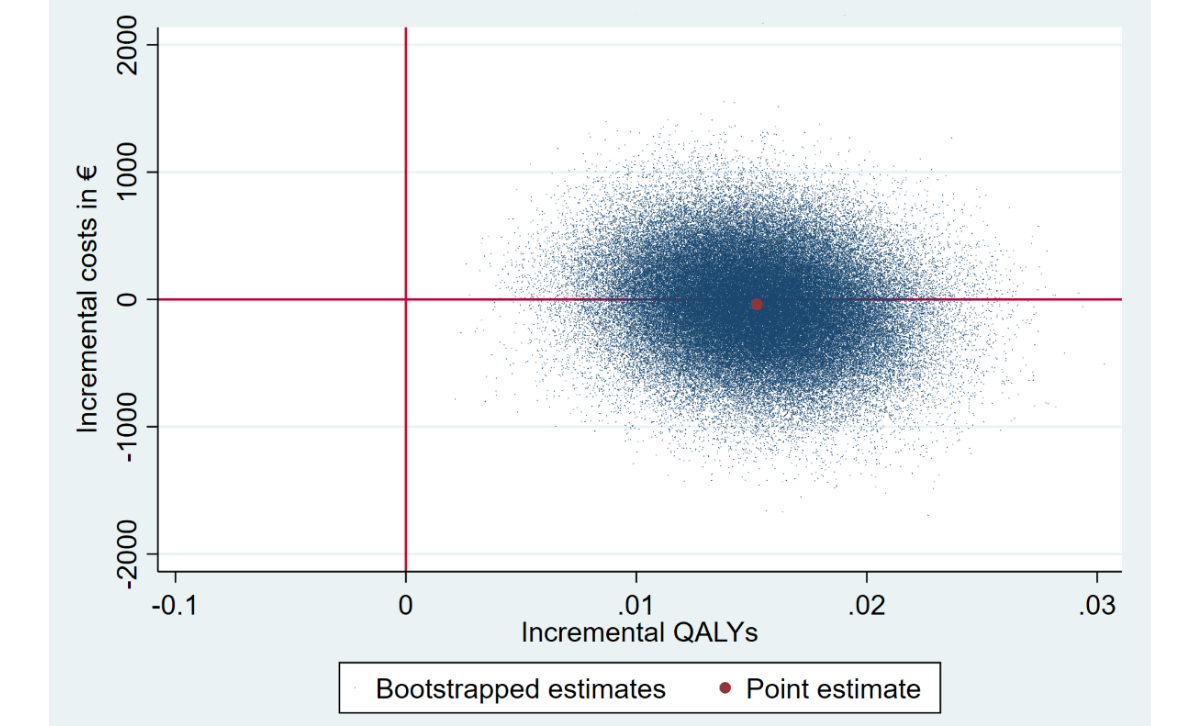
Scatterplot of 5000 replicates of the incremental cost-effectiveness ratio (mean differences in costs and QALYs) on the cost-effectiveness plane from the societal perspective: iSMI versus WLC. QALYs: quality-adjusted life years.

**Figure 4:**
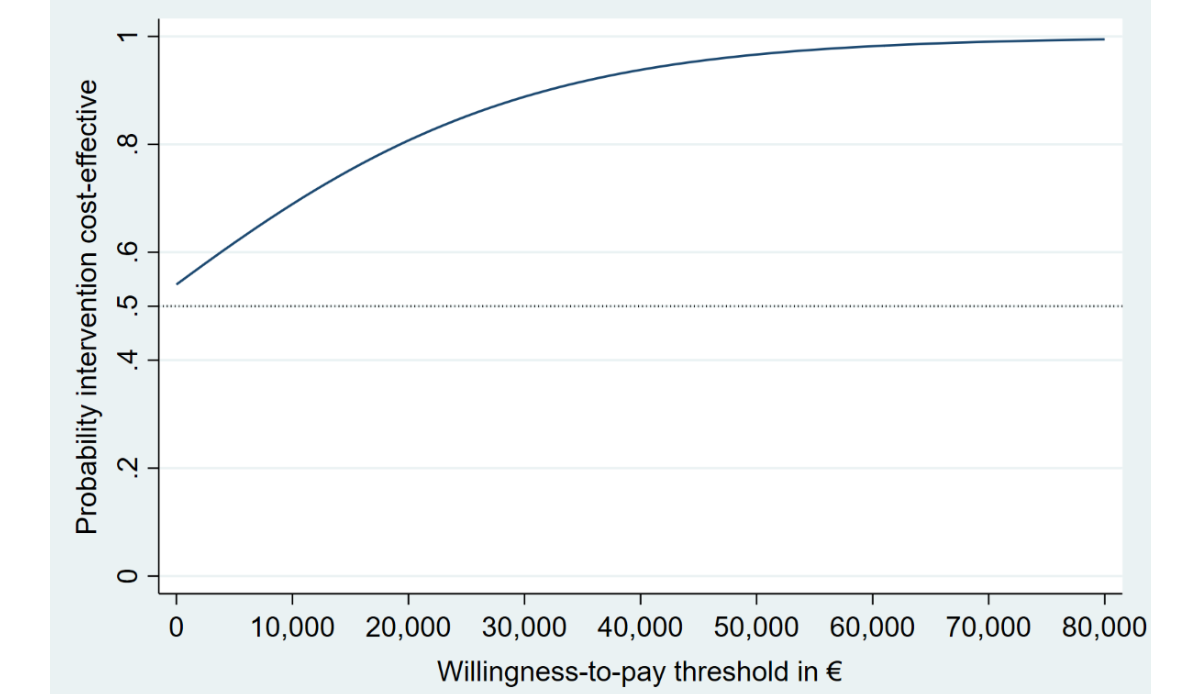
Cost-effectiveness acceptability curves (QALYs) from the societal perspective. QALYs: quality-adjusted life years.

Cost-utility analysis revealed similar results (Table 2). Again, the iSMI was likely to be dominant relative to the WLC with more QALY gains at lower costs, resulting in a 55% probability that the iSMI is cost-effective at a societal willingness-to-pay threshold of €0 (Figure 3). This probability increased to 80% at a societal willingness-to-pay per QALY gained of €20,000 (US $21,938.20; Figure 4). Using NBRF, no subgroups were identified for which iSMI was significantly more (or less) cost-effective (all *P*-values ≥.05 for both WTPs of 0€ and €20,000 [US $21,938.20] per QALY gained, respectively).

#### Employer’s Perspective

The iSMI condition showed a net benefit per participant of €76 (£65; ie, US $83.37; 95% CI €−498 to 665 [ie, 95% CI US $–546.26 to $729.45]) and a benefit-to-cost ratio of 1.77 (95% CI €−4.03 to 7.72 [ie, 95% CI US $–4.42 to $8.47]). The ROI was 77% (95% CI −503% to 672%), respectively. The probability of a positive ROI was 78% for the iSMI condition (Table 3).

**Table 3:** Results from the employer’s perspective (main and sensitivity analyses) of adjusted cost-benefit analyses based on 5000 bootstrapped linear regression models.

			Costs^a^					Benefits^b^					Financial returns								
			Total		95% CI		Total			95% CI		NB^c^			95% CI		ROI^d^ (%)		95% CI		P^e^ (%)
**Main analysis**																					
	Unguided intervention	99		—^f^		175			−399 to 764		76			−498 to 665		77		−50 to 672		78	
**Sensitivity analysis 1^g^**																					
	Unguided intervention	99		—		217			−226 to 687		118			−325 to 588		120		−329 to 594		96	
**Sensitivity analysis 2^h^**																					
	Unguided intervention	299		—		175			−399 to 764		−124			−698 to 465		−41		−233 to 156		14	

^a^Includes intervention costs.

^b^Benefits are the difference in productivity costs between the intervention group and the control condition.

^c^NB: Net benefit linear regression models adjusted for baseline costs due to absenteeism and presenteeism.

^d^ROI: Return on investment linear regression models adjusted for baseline costs due to absenteeism and presenteeism.

^e^Probability of positive return on investment.

^f^Not available.

^g^Sensitivity analysis 1 analyses for winzorizing cost outlier to 95% percentiles.

^h^Sensitivity analysis 2 analyses adding intervention costs of €299 (ie, US $ 327.98; instead of €99 [US $108.59]).

#### Sensitivity Analyses

The results of the sensitivity analyses are summarized in Table 2 and Table 3. Winsorizing cost outliers led to a slightly higher probability that the intervention produces higher health gains at lower costs than WLC with regard to symptom-free status (66%) and QALYs (65%) at a societal WTP threshold of €0. The net benefit increased when cost outliers were winzorized, and the probability of a positive return on investment was 96% for the iSMI. Increasing intervention costs up to 3 times (€299 [US $327.98] instead of €99 [108.59]), this probability decreased to 33% regarding symptom-free status and QALYs gained at a WTP of €0. However, at a WTP of €20,000 [US $21,938.20] per QALY gained, the probability of being cost-effective was comparable to the main analysis (eg, 78%). Return on investment became negative when intervention costs were increased up to 3 times, and the probability of a positive financial return decreased to 14% for the iSMI. Using the EQ-5D-3L resulted in a probability of 55% regarding QALYs gained at a WTP of €0, identical to the main analysis.

## Discussion

### Principal Findings

This study evaluated the cost-effectiveness and cost-utility of a universal unguided digital stress management intervention for employees from a societal perspective and the cost-benefit from the employer’s perspective compared with a waitlist control condition over a 6-month time horizon. From a societal perspective, the iSMI had a high probability of being cost-effective (eg, 80% at a WTP of €20,000 [US $21,938.20] per QALY gained). From an employer’s perspective, the iSMI had a high probability of a positive return on investment with 78%.

### Comparison With Previous Work

Evidence for economic evaluations of universal digital prevention of mental disorders is scarce. To our knowledge, this is the first economic evaluation of a universal iSMI to reduce stress in employees using a societal and an employer’s perspective. The results of our study are in line with other economic evaluations of the same iSMI for stressed employees in the field of indicated prevention (PSS≥22) in which e-coaches provided personalized feedback throughout the intervention [15]. Kählke et al [15] demonstrated that more QALYs were generated for lower costs in the guided iSMI compared with a waitlist control condition, indicating a similar probability of 76% compared with our study that the intervention was cost-effective compared with WLC at a societal WTP of €20,000 [US $21,938.20] per QALY gained. A similar probability of 71% at a societal WTP of US $25,000 was also found by Lindsäter et al [33], who evaluated therapist-guided internet-based Cognitive Behavioral Therapy for stress-related disorders compared to WLC.

Results from the employer’s perspective are comparable to the study by Ebert et al [16], who examined the cost-benefit of the same iSMI as Kählke et al [15]. The analysis of Ebert et al [16] yielded a similar benefit-to-cost ratio of 1.6 (95% CI €−1.2-4.5 [US $–1.32 to $4.94]) compared with a benefit-to-cost ratio of 1.77 (95% CI €−4.0 to 7.7 [US $–4.39 to $8.45]) in this study. Our results from the employer’s perspective are also in line with findings from a recent systematic review indicating that addressing mental health in employees improves both their well-being and productivity [34]. Regarding the ROI analyses, our findings compare favorably to a systematic review of face-to-face health promotion interventions at the workplace (12 RCTs) that showed, on average, a negative ROI (ROI=–0.22, 95% CI 0.27-0.16; min=−4.3, max=5) [35]. van Dongen et al [36] also showed a negative return on investment of a combined social and physical environmental intervention in office employees.

### Limitations

The following limitations have to be considered. First, contrary to the pharmaco-economic guidelines recommending TAU as a comparator [37], the iSMI was compared with a wait-listed control condition, albeit with unrestricted access to usual care. Second, due to the limited time horizon of 6 months, longer-term costs and effects caused by chronic stress (eg, the onset of a new health disorder or staff turnover) could not be analyzed. Future studies should examine whether treatment effects and costs are sustained over a longer period of time or decline. Third, preference-based utility values were only evaluated at baseline and after 6 months, and thus, an immediate treatment effect was not assessed. Fourth, costs due to presenteeism were only assessed using the Osterhaus method. However, this method tends to overestimate costs because it assumes a 1:1 relationship between work hours lost and productivity losses, but that relationship might be better described as an elastic relationship, for example, 100% to 90%. Future studies should also include alternative methods (eg, Health and Labor Questionnaire method) to calculate costs due to presenteeism. Fifth, self-report questionnaires were used, which may have led to effects related to social desirability and response bias [38]. Future studies could consider claims data from health insurance companies. Sixth, the sample is characterized by a high proportion of female (302/396, 76.3%) as well as highly educated (285/396, 72%) participants, limiting the generalizability of the study findings. However, women with higher education are often the typical target group who take part in preventive internet-based interventions [39].

### Clinical Implications

Evidence-based recommendations on the cost-effectiveness of interventions can help inform decision-makers from the societal and employer’s perspective when choosing preventive stress interventions. The findings of this study support the hypothesis that a universal prevention approach could be a cost-effective strategy to reduce the adverse consequences of work-related stress besides its shown effectiveness [14]. However, it must be taken into account that the participants in the study already had a rather high initial stress level on average. That is, we cannot say whether the cost-effectiveness results will hold up if increased numbers of participants with low levels of stress participate in the intervention. An unguided universal iSMI might be appropriate from an economic perspective since there are no costs for screening compared with an iSMI for indicated prevention and additionally no costs for therapeutic guidance compared with guided interventions. Since health care professionals and available resources are often limited, unguided interventions have the potential to be implemented in routine occupational care on a large scale. Meta-analytic evidence shows that unguided interventions generate similar effects compared with guided interventions in individuals with low symptom severity [40]. However, the evidence on this is heterogeneous [41]. Therefore, future studies should directly compare the cost-effectiveness of guided compared with unguided iSMIs. From a health economic point of view, no moderators were revealed in NBRF analyses. However, this could also have been due to the small sample size and should be further researched in an individual participant data meta-analysis in the future.

### Conclusions

A universal unguided digital stress management intervention appears to be cost-effective (ie, the health effects achieved represent good value for the invested money) as well as offering a favorable cost-to-benefit ratio (ie, the financial gains outweigh the intervention costs, so the return on investment is positive). Further research is needed to examine the long-term effects on cost-effectiveness and to compare digital stress management intervention with standard care. Unguided digital stress prevention carries the promise of being scalable, which would leverage the cost-effectiveness and positive return on investment of this type of universal stress prevention in the working population.

## Appendix

Multimedia Appendix 1 CHEERS (Consolidated Health Economic Evaluation Reporting Standards) checklist.

## Abbreviations

AQoL-8D: Assessment of Quality of Life
AUC: area under the curve
CEA: cost-effectiveness analysis
CEAC: cost-effectiveness acceptability curve
CUA: cost-utility analysis
ICER: incremental cost-effectiveness ratio
IG: intervention group
iSMI: internet-based stress management intervention
MICE: multiple imputations by chained equations
NB: net benefit
NBRF: net-benefit regression framework
PSS: Perceived Stress Scale
QALY: quality-adjusted life year
RCT: randomized controlled trial
ROI: return-on-investment
SURE: seemingly unrelated regression equations
TAU: treatment as usual
TiC-P: Treatment Inventory of Costs in Patients with Psychiatric Disorders
WLC: waitlist control condition
WTP: willingness-to-pay

## Data Availability

The datasets generated during and/or analyzed during this study are not publicly available but are available from the corresponding author on reasonable request by qualified scientific and medical researchers for legitimate research purposes. The data will be shared under a data-sharing agreement once the results of the health-economic evaluation are published. Requests should be sent to the last author.
